# Performances of machine learning algorithms in discriminating sacroiliitis features on MRI: a systematic review

**DOI:** 10.1136/rmdopen-2023-003783

**Published:** 2023-11-23

**Authors:** Sun Jae Moon, Seulkee Lee, Jinseub Hwang, Jaejoon Lee, Seonyoung Kang, Hoon-Suk Cha

**Affiliations:** 1 Department of Medicine, Santa Marie 24 Clinic, Seongnam-si, Korea (the Republic of); 2 Department of Medicine, Samsung Medical Center, Sungkyunkwan University School of Medicine, Seoul, Korea (the Republic of); 3 Department of Data Science, Daegu University, Gyeongsan-si, Korea (the Republic of)

**Keywords:** Machine Learning, Spondylitis, Ankylosing, Magnetic Resonance Imaging, Epidemiology

## Abstract

**Objectives:**

Summarise the evidence of the performance of the machine learning algorithm in discriminating sacroiliitis features on MRI and compare it with the accuracy of human physicians.

**Methods:**

MEDLINE, EMBASE, CIHNAL, Web of Science, IEEE, American College of Rheumatology and European Alliance of Associations for Rheumatology abstract archives were searched for studies published between 2008 and 4 June 2023. Two authors independently screened and extracted the variables, and the results are presented using tables and forest plots.

**Results:**

Ten studies were selected from 2381. Over half of the studies used deep learning models, using Assessment of Spondyloarthritis International Society sacroiliitis criteria as the ground truth, and manually extracted the regions of interest. All studies reported the area under the curve as a performance index, ranging from 0.76 to 0.99. Sensitivity and specificity were the second-most commonly reported indices, with sensitivity ranging from 0.56 to 1.00 and specificity ranging from 0.67 to 1.00; these results are comparable to a radiologist’s sensitivity of 0.67–1.00 and specificity of 0.78–1.00 in the same cohort. More than half of the studies showed a high risk of bias in the analysis domain of quality appraisal owing to the small sample size or overfitting issues.

**Conclusion:**

The performance of machine learning algorithms in discriminating sacroiliitis features on MRI varied owing to the high heterogeneity between studies and the small sample sizes, overfitting, and under-reporting issues of individual studies. Further well-designed and transparent studies are required.

**WHAT IS ALREADY KNOWN ON THIS TOPIC**

- There are some systematic reviews on the accuracy of human physicians’ diagnosis of sacroiliitis using MRI.
- Although machine learning has been developed for about a decade, the accuracy of diagnosing sacroiliitis using this technology has not yet been published in systematic reviews.

**WHAT THIS STUDY ADDS**

- We concluded that the area under the curves of these machine learning algorithms in discriminating sacroiliitis features on MRI ranged from 0.76 to 0.99, and their sensitivity and specificity ranged from 0.56 to 1.00 and 0.67 to 1.00, respectively.
- Whereas the sensitivity and specificity of human doctors in the same cohort ranged from 0.67 to 1.00 and 0.78 to 1.00, respectively.
- We found some limitations in the studies reviewed, including problems with small sample sizes, overfitting and under-reporting issues.

**HOW THIS STUDY MIGHT AFFECT RESEARCH, PRACTICE OR POLICY**

- We encourage future researchers to transparently report and perform well on study designs.
- We also recommend peer reviewers and future researchers to adherence to artificial intelligence-specific reporting and review guidelines.

## Introduction

In the last decade, diagnostic imaging has enabled disease activity and structural damage to be measured non-invasively, enabling early diagnoses in clinical practice.^1^ With the establishment of the Assessment of Spondyloarthritis International Society (ASAS) criteria for the classification of spondyloarthritis (SpA) in 2009, the utility of MRI increased.^2^ MRI has become a valuable imaging modality for early diagnoses in the preradiographic phase and identification of active sacroiliac joint inflammation.^3^ According to a survey of European Alliance of Associations for Rheumatology (EULAR) member countries, the most common reason for MRI use is to diagnose or manage sacroiliitis and spondylosis; however, only 10% of countries have MRI training in their rheumatologists’ training curricula.^4^ In addition, a recent systematic review reported that a human physician’s diagnosis of axial SpA using MRI features is highly variable across studies.^5^ Moreover, these MRI readings require time and experience from both rheumatologists and radiologists.^6^

Owing to this unmet need, artificial intelligence (AI) is being actively developed for musculoskeletal imaging and used in areas such as automated image analysis and reading.^7^ In particular, similar to the concept of ‘omics’ in biology, a field of data science called ‘radiomics’ has recently emerged in specialised imaging devices such as CT and MRI to help diagnose based on the complexity and connectivity of pixel-level components that are difficult for radiologists to see.^8^ Therefore, the role of machine learning is becoming more critical.^9^ This AI subtype learns, trains and conducts classes on behalf of human doctors to process and interpret radiomic information in MRI. Machine learning assesses performances through validation, internal test and external test processes. The validation process tunes the parameters and constructs a suitable model. An example of a validation method is cross-validation. The k-fold cross-validation method randomly divides the data into k-folds and uses one for testing and the other as a training dataset.^10^ The model may undergo another process, the internal test process.^11^ External validation involves testing the model in a completely independent cohort.^11^

Over the past decade, many machine learning algorithms have been developed, validated and published in various fields of rheumatology; however, they have been criticised for being limited to experimental development on small samples.^12^ In addition, a systematic review of the methodology of supervised machine learning algorithm studies concluded that the performance of the machine learning algorithm is likely to be overestimated because of small samples, an overfitting risk and few external validation studies.^13 14^ Our study aims to summarise the performances of machine learning algorithms in discriminating sacroiliitis features on MRI and compare them with human physicians, providing critical appraisal for the performances of machine learning algorithms.

## Methods

This systematic review was performed in accordance with the Preferred Reporting Items for Systematic Reviews and Meta-Analyses guideline.^15^

## Search strategy and selection procedure

We used MEDLINE, EMBASE, Cumulative Index to Nursing and Allied Health Literature (CIHNAL), and Web of Science as the main core databases, and conference proceedings (meeting abstracts) from IEEE Xplore, American College of Rheumatology (ACR), and European Alliance of Associations for Rheumatology (EULAR) archives as additional databases. The search strategies and queries were designed using keywords and subject terms related to the following key phrases: sacroiliac joint or sacroiliitis, machine learning and performance. These were then reviewed for appropriateness by librarians. The search was conducted on 4 June 2023 and the language of the publications was unrestricted. Publication dates were limited to 1 January 2008, which is 1 year before the ASAS classification of axial SpA was first published.^16^ The criteria for inclusion in the review were that the studies were based on adult patients with MRIs that included both sacroiliac joints, used a machine learning algorithm on MRI to determine the presence or absence of sacroiliitis features, and presented quantitative performance metrics. The ideal scenario involves patients with axial SpA and non-specific low back pain on sacroiliac MRI. However, we did not limit ourselves to a specific patient population as we suspected that the research could be diverse. Machine learning refers to algorithms that have an inherent process of determining answers through experience and algorithms that determine optimal solutions through a training process.^17^ In addition, because of the wide variety of performance metrics for machine learning approaches, we included all types of performance indices, including metrics expressed in numerical terms, as defined by the authors of the studies. Representative performance indices include sensitivity, specificity, accuracy, recall, precision, F1-score, Mathew’s correlation and area under the curve (AUC), which are used to predict the power of the machine learning method.^10^ Before study selection, we created a checklist of predefined eligibility and exclusion criteria for titles and abstracts independently checked by two authors (SJM and SL). The matching process was repeated until all studies were fully matched. Discrepancies involving the study design or statistical issues were resolved by one author (JH), a statistical expert, and the clinical aspects were resolved by another author (H-SC), a clinical expert; in some cases, the opinions of both experts were pooled to resolve these discrepancies.

### Data extraction procedure

First, we extracted information on the publication type and funding source or sector. Sociodemographic variables such as age, gender and country were extracted. The distribution of disease groups (eg, axial SpA) and information about sacroiliitis (eg, medication information and disease activity or duration), if any, were extracted and organised in a free-text format, and then categorised by commonalities. The MRI sequences of each study and other MRI performance-related metrics were extracted, if reported. The machine learning algorithm information and details were collected according to the Checklist for Artificial Intelligence in Medical Imaging (CLAIM).^18^ In particular, the algorithm model type, whether internal or external validation was performed, the region of interest (ROI) extraction method and ground truth information were extracted. The reported performance indices (eg, sensitivity and specificity) were extracted according to the type and evaluation process (validation, internal test, or external test). Studies comparing machine learning and human doctor performances were extracted separately. Before extraction, the items were listed and coded on a Microsoft Excel spreadsheet. The entire process was performed independently by two authors (SJM and SL), and any discrepancies were resolved until the studies included were fully matched.

### Quality assessment

We used the Prediction Model Risk of Bias Assessment Tool (PROBAST) as the quality assessment tool. This tool was developed to evaluate the risk of bias (RoB) in participants, predictors, outcomes and analyses based on signalling questions.^19^ However, because this tool is primarily an evaluation method for prediction tools using regression, directly applying it to our studies’ machine learning algorithms was challenging; therefore, two signalling questions (4.5 and 4.9) were removed.^13^ The quality assessment results were summarised as a percentage of the overall RoB for each domain^19^ and were performed independently by two authors (SJM and SL). For discrepancies between independent assessments, the same process was repeated, and consensus was reached through further discussion between the two authors (SJM and SL).

### Evidence summary

To determine sensitivity and specificity, we performed a meta-analysis of bivariate random effects. However, significant statistical heterogeneity was observed; therefore, we did not attempt to present the pooled estimates from the meta-analysis.^20^ Instead, the variables and performance metrics of the studies were organised in a tabular view and then subgrouped by the variables responsible for heterogeneity, and the sensitivity and specificity metrics were organised in a forest plot to provide a visual distribution. All statistical estimates are provided with 95% CI. Forest plots were drawn using Review Manager (RevMan) (V.5.4. The Cochrane Collaboration, Oxford, UK).

## Results

In total, 1882 studies were retrieved from the four core databases, and 499 studies were obtained from additional databases, totalling 2381. After removing duplicates and reviewing titles and abstracts, 26 studies were selected for full-text review. Of these, 16 were removed after reading the full text, leaving ten studies^21–30^ for inclusion in the final review (online supplemental figure 1). Online supplemental table 1 presents the details of the search queries and number of searches per database.

10.1136/rmdopen-2023-003783.supp1 Supplementary data

### General characteristics of the included studies

Of the 10 included studies, 8 studies^21–23 25 26 28–30^ were published as original articles and 2 studies^24 27^ as conference proceedings. Among these studies, four^21 24 27 29^ were from Europe, three^22 25 28^ from Asia and two^23 30^ from Latin America. The other one study, Bressem *et al*^26^ was from multiple continents. The data sources of six studies^22–24 28–30^ were based on patients in the hospital, and the remaining four^21 25–27^ were based on patients in cohorts already built from other studies. Except for three studies^24 27 30^ that did not report information on sex and age, the remaining studies had a range of 40%–71% males, and the mean age was under 40 years in four studies and over 40 years in the other three studies. Short tau inversion recovery MRI sequences were analysed in five studies.^21 23 25 27 30^ Table 1 and online supplemental table 2 provide more details.

**Table 1:** General characteristics of the studies included (10 studies)

	Publication type	Country	Database	Sex, male/total (%)	Mean age (categorised by 40 years)	MRI sequence
Bordner *et al*^21^	Original article	France	DESIR (internal) ASAS (external)	126/256 (49) (23/47 (49) for the external cohort)	<40 years for the internal and the external cohort	STIR and T1 weighted
Ye *et al*^22^	Original article	China	Hospital	422/638 (66)	<40 years	SPAIR
Tenório *et al*^23^	Original article	Brazil	Hospital	29/46 (63)	>40 years	STIR and SPAIR
Roels *et al*^24^	Conference proceeding	Belgium	Hospital	Not reported	Not reported	T1 and T2 weighted
Lin *et al*^25^	Original article	China	Hong-Kong multicentre cohort	218/388 (56)	>40 years	STIR and T1 weighted
Bressem *et al*^26^	Original article	Multinational*	GESPIC and OptiRef (internal) ASAS (external)	245/477 (51)(46/116 (40) for the external cohort)	<40 years for the internal and the external cohort	Fluid-sensitive fat-suppressed and T1 weighted
Nicolaes *et al*^27^	Conference proceeding	Belgium	C-axSpAnd and BE MOBILE RCT	Not reported	Not reported	STIR and T1 weighted
Lee *et al*^28^	Original article	South Korea	Hospital	56/79 (71)	<40 years	Gadolinium-enhanced fat-suppressed and T1 weighted
Kepp *et al*^29^	Original article	Switzerland	Hospital	40/90 (44)	>40 years	TIRM and T1 weighted
Faleiros *et al*^30^	Original article	Brazil	Hospital	Not reported	Not reported	STIR and SPAIR

*Europe, China, Taiwan, Turkey and Colombia.

ASAS, Assessment of Spondyloarthritis International Society; DESIR, DEvenir des Spondyloarthropathies Indifferenciees Recentes; GESPIC, German Spondyloarthritis Inception Cohort; MOBILE, Evaluation of the efficacy and safety of bimekizumab in subjects with active non-radiographic axial spondyloarthritis; OptiRef, Optimal Referral Strategy for Early Diagnosis of Axial Spondyloarthritis; SPAIR, SPectral Attenuated Inversion Recovery; STIR, Short τ Inversion Recovery; TIRM, Turbo Inversion Recovery Magnitude.

### Technical details of machine learning algorithm

Six^22 23 27–30^ of the 10 studies included manually extracted ROIs, except for Bressem *et al*^26^ who did not undergo ROI extraction. Regarding classification models, six^21 24–28^ of the included studies used deep learning techniques, whereas the remaining four studies^22 23 29 30^ developed non-deep learning algorithms. Of the 10 studies, 8^22–24 26–30^ reported the validation process performance, 3^21 25 30^ showed the internal testing process performance and 3^21 24 26^ presented the external testing process performance. Seven studies^21 23 25 26 28–30^ set ground-truth annotation as the presence or absence of ASAS features on the MRI. Two conference studies^24 27^ focused on the presence or absence of bone marrow oedema (BMO), and Lin *et al*^25^ defined BMO as the diagnosis of axial SpA (see table 2 for details).

**Table 2:** Technical details of machine learning algorithms (10 studies)

	Extracting ROI	Classification models	Methods of evaluating performance	Ground truth annotations
Bordner *et al*^21^	Automatically	DL: Mask-RCNN	Internal and external test	ASAS MRI sacroiliitis±of each persons’ MRI
Ye *et al*^22^	Manually (two MS radiologists)	mRMR and LASSO	Validation	ASAS clinical diagnosis of AxSpA±of each person
Tenório *et al*^23^	Manually (two MS radiologists)	ANN	Validation	ASAS MRI sacroiliitis±of each persons’ MRI
Roels *et al*^24^	Automatically	DL: ResNet18	Validation and external test	BMO±of each persons’ MRI
Lin *et al*^25^	Automatically	DL: CNN (+ Attention-U-Net)	Internal test	ASAS MRI sacroiliitis±of each persons’ MRI or each images
Bressem *et al*^26^	Not applicable*	DL: Res-Neural network	Validation and external test	ASAS MRI sacroiliitis ±, structural changes of SI joints ±, active inflammatory changes of SI joints±of each persons’ MRI
Nicolaes *et al*^27^	Manually (three MS radiologists)	DL: unclear architecture	Validation	BMO±of each persons’ MRI
Lee *et al*^28^	Manually (two rheumatologists and one radiologist)	DL: CNN (+ResNet18)	Validation	ASAS MRI sacroiliitis±of each persons’ MRI or each images
Kepp *et al*^29^	Manually (two radiologists)	ANN (+k-nearest neighbour)	Validation	ASAS MRI sacroiliitis±, TIRM positive ASAS sacroiliitis±, MRI sacroiliitis versus degenerative changes of each persons’ MRI
Faleiros *et al*^30^	Manually (one MS radiologist)	ANN, SVM, k-nearest neighbour	Validation and internal test	ASAS MRI sacroiliitis±of each persons’ MRI

*Not extracted ROI.

ANN, artificial neural network; ASAS, Assessment of Spondyloarthritis International Society; AxSpA, axial spondyloarthritis; BMO, bone marrow oedema; CNN, convolutional neural network; DL, deep learning; LASSO, least absolute shrinkage and selection operator; mRMR, minimum-redundancy-maximum-relevance; MS, muscular skeleton; RCNN, regions with convolutional neural networks; ROI, region of interest; SI, sacroiliac; SVM, support vector machine; TIRM, Turbo Inversion Recovery Magnitude.

### Performance of machine learning algorithms and comparison with humans

#### Validation process

**Figure 1:**
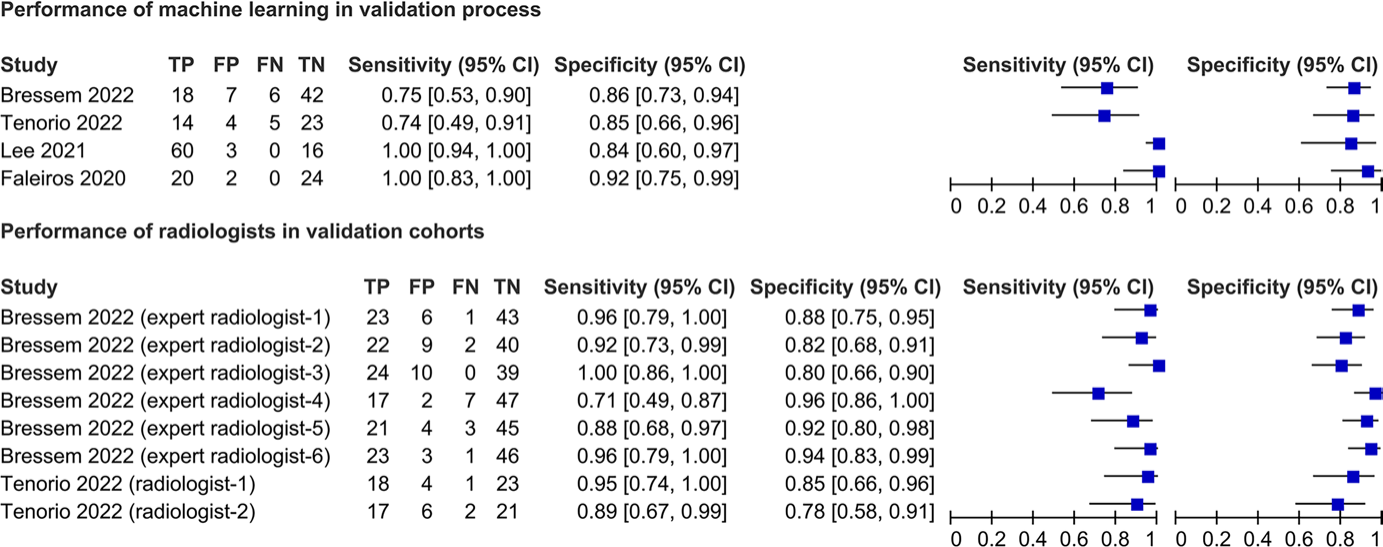
Performance of machine learning algorithms in the validation process and comparison with humans. TP, true positive; FP, false positive; FN, false negative; TN, true negative.

Eight studies^22–24 26–30^ reported the validation process performances. These studies reported AUCs ranging from 0.77 to 0.99 (online supplemental table 3). Of these studies, five^23 26 28–30^ used the presence/absence of ASAS MRI sacroiliitis features as the ground truth (online supplemental table 3). Four of these studies^23 26 28 30^ were presented as forest plots, except for one study (Kepp *et al*^29^), which did not report the sensitivity or specificity (figure 1). The sensitivity of the machine learning method ranged from 0.74 to 1.00, and the specificity ranged from 0.84 to 0.92 (figure 1). In the same cohort, radiologists exhibited sensitivity ranging from 0.71 to 1.00 and specificity ranging from 0.78 to 0.96 (figure 1). Online supplemental table 3 provides more details.

#### Internal test process

**Figure 2:**
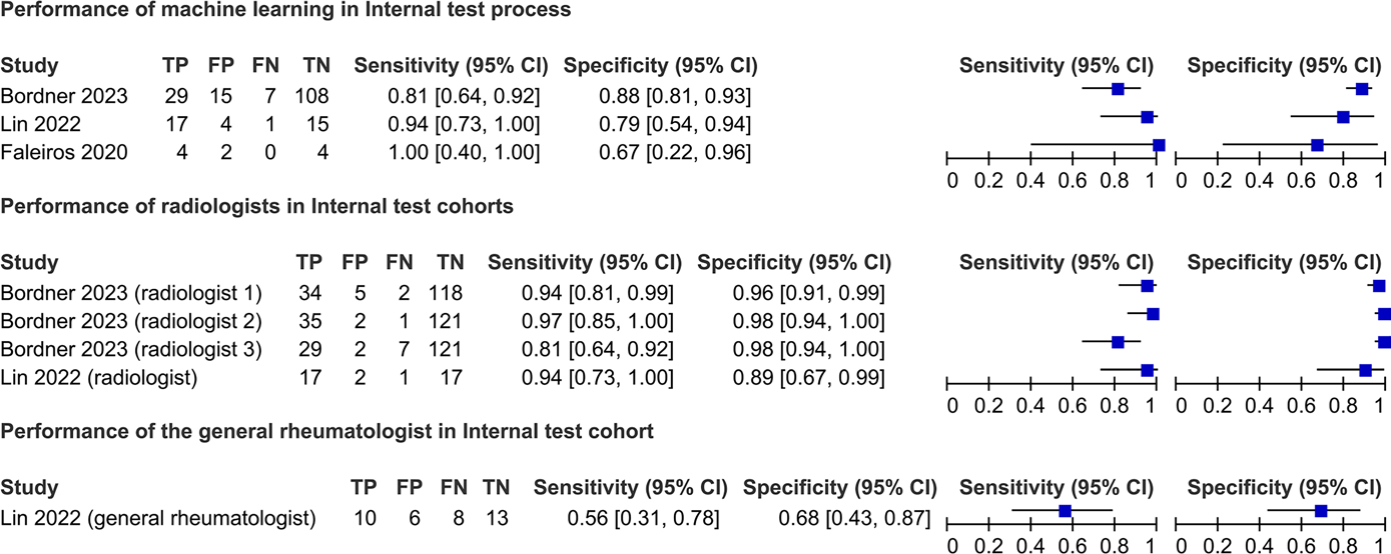
Performance of machine learning algorithms in the internal test process and comparison with humans. TP, true positive; FP, false positive; FN, false negative; TN, true negative.

Three studies^21 25 30^ reported the internal test process performances, with AUCs ranging 0.80–0.98 (online supplemental table 4). These studies used the same ground truth standard (ASAS MRI sacroiliitis), and the sensitivity and specificity distributions were visually presented as forest plots (figure 2). The machine learning sensitivity ranged from 0.81 to 1.00. The specificity ranged from 0.67 to 0.88; radiologists under the same conditions exhibited a sensitivity of 0.81–0.97 and specificity of 0.89–0.98 (figure 2). The general rheumatologist reported by Lin *et al*^25^ had a sensitivity of 0.56 and specificity of 0.68 (figure 2). Online supplemental table 4 provides more details.

#### External test process

**Figure 3:**
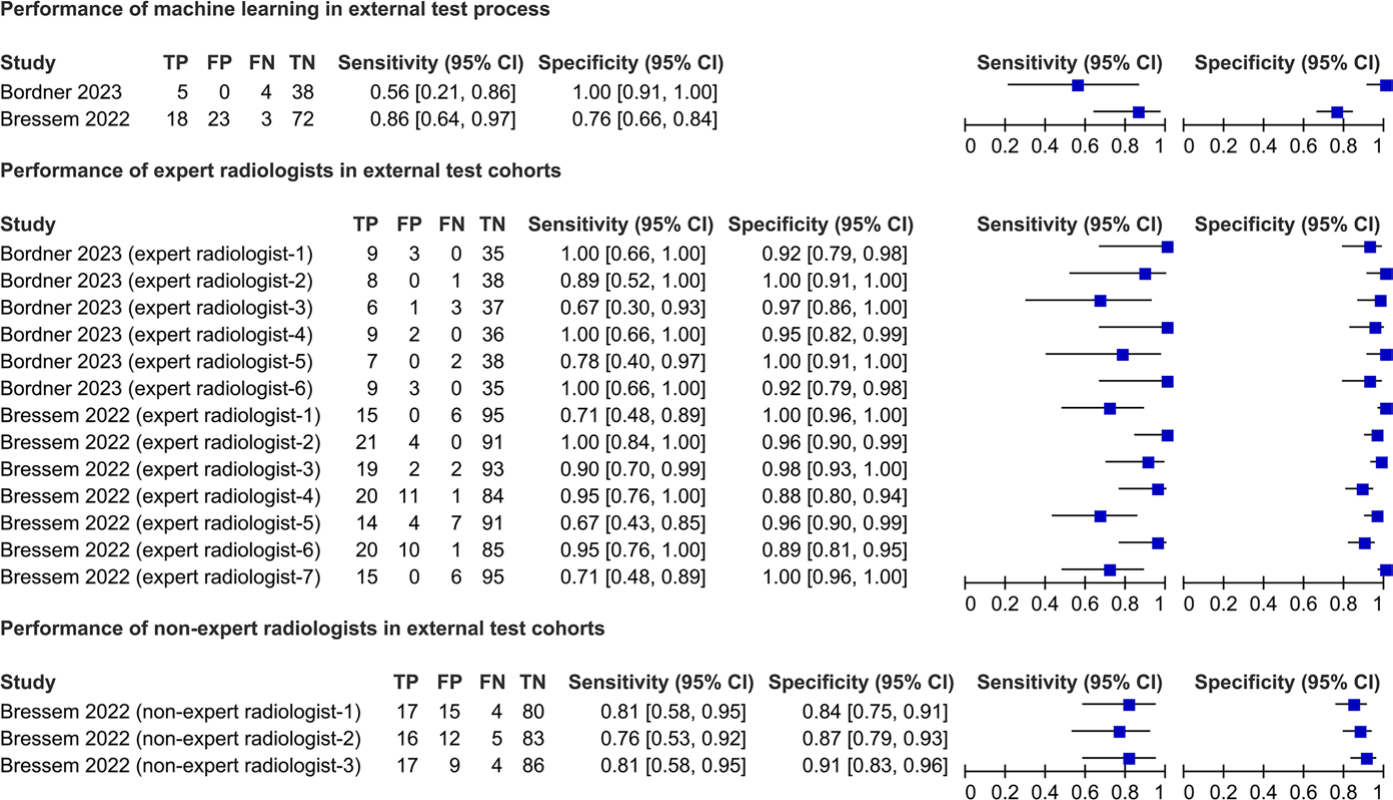
Performance of machine learning algorithms in the external test process and comparison with humans. TP, true positive; FP, false positive; FN, false negative; TN, true negative.

Three studies^21 24 26^ presented the external testing process performance, and the AUC values reported by all studies ranged from 0.76 to 0.94 (online supplemental table 5). Two studies^21 26^ used a common ground truth (ASAS MRI sacroiliitis). Their machine learning algorithms exhibited sensitivities of 0.56 and 0.86, and specificities of 1.00 and 0.76 (figure 3). In the same setting, expert radiologists exhibited a sensitivity ranging from 0.67 to 1.00, and a specificity ranging from 0.88 1.00 (figure 3). In contrast, non-expert radiologists exhibited a sensitivity ranging from 0.76 to 0.81, and a specificity ranging from 0.84 to 0.91 (figure 3). Online supplemental table 5 provides more details.

### Quality assessment

**Figure 4:**
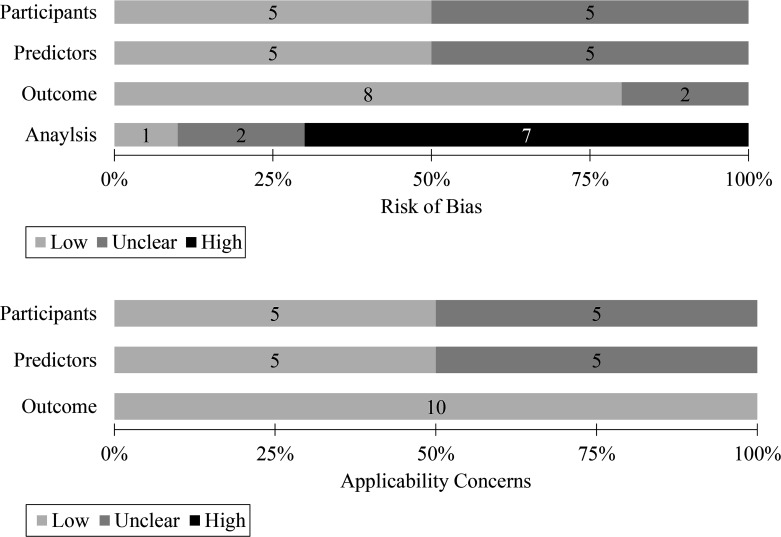
Summary of quality assessment (Prediction Model Risk of Bias Assessment Tool).

The participant and predictor domains were rated ‘low’ and ‘unclear’ for half of the studies^21 22 25 26 29^ in terms of the RoB and applicability concern (figure 4). The outcome domain was graded ‘low’ for eight studies,^21–23 25 26 28–30^ two conference proceedings studies^24 27^ were ‘unclear’, and all studies were classified as ‘low’ in terms of the applicability concern (figure 4). However, the analysis domain in the RoB scored ‘high’ because seven studies^21–23 25 28–30^ had small samples or performed an internal validation without using a cross-validation or bootstrapping technique; two studies^24 27^ were classified as ‘unclear’, and the study by Bressem *et al*^26^ was rated as ‘low’ (figure 4). See online supplemental table 6 for detailed domain-specific and study-specific judgements.

## Discussion

This systematic review summarises the evidence on the performances of machine learning algorithms in discriminating sacroiliitis MRI features. Over half of the studies developed deep learning models, used the ASAS sacroiliitis criteria as the ground truth, and extracted the ROIs manually. All studies presented performances in terms of the AUC, and many exhibited performances in terms of the accuracy, sensitivity and specificity. The predictive power of the machine learning algorithms can be summarised as an AUC of 0.76–0.99, sensitivity of 0.56–1.00 and specificity of 0.67–1.00. In comparison, radiologists under similar conditions exhibited a sensitivity of 0.67–1.00 and specificity of 0.78–1.00. However, the sensitivity and specificity distribution results showed that both machine learning methods and human doctors exhibited a wide range of sensitivity and specificity, suggesting heterogeneity between studies.

The main reasons for the heterogeneity in the performances across the studies are differences in the ground truth, clinical aspects of the datasets, and architectures of the machine learning models. The models of the studies included are primarily based on a human definition of the ground truth of MRI sacroiliitis discrimination factors (supervised model), and the performances of the machine learning algorithms are inevitably affected by the performance characteristics of human doctors. Our results show variation in radiologists’ performances in the same cohort and setting, and is supported by a previous systematic review on the performance of human doctors in diagnosing axial SpA using MRI features. This study reported sensitivity ranging from 0.35 to 0.91 and specificity ranging from 0.75 to 0.90, with a wide range in the sensitivity and specificity and high heterogeneity among the studies.^5^ Heterogeneity was reported to be caused by variations in the clinical characteristics and diagnostic criteria among individual studies,^5^ and the studies included in our review involved similar characteristics. The proportion of women and mean age varied across studies (table 1). The machine learning algorithms and ground truths varied across studies (table 2). However, only five studies reported other clinical characteristics, such as symptom duration and biological markers of disease activity, which may contribute to the heterogeneity. Even within these studies, the illness duration varied widely, from 1.6 to 11.6 years (online supplemental table 2). In terms of the machine learning model, the advent of newer techniques such as those involving deep learning, in conjunction with the performance of traditional algorithms, has unfavourably led to heterogeneity between studies (table 2).

The quality of the individual studies was limited. In the analysis domain of the PROBAST quality assessment tool, most studies in this review were rated as having a high bias risk owing to the small sample sizes and overfitting problems (figure 4). Although the relatively rare incidence of rheumatology has few patients, this is a common criticism in other machine learning systematic reviews.^13^ In addition, an external test should be conducted with an independent population to overcome overfitting issues,^11^ which was only conducted in three studies.^21 24 26^ In addition, the MRI quality affects the performance of the machine learning algorithms,^31^ which was not reported in over half of these studies. However, when different MRI machines were used at various multinational centres, Bressem *et al*^26^ reported detailed information on the MRI quality indices. Performance reporting is recommended for all data partitions^18^ with the limitation that the studies included in this review selectively reported each evaluation process (table 2). A detailed description is required to reproduce this model.^18^ In some cases, such as that reported by Tenório *et al,*^23^ the algorithm code was published on an open-source webpage, which makes understanding and reproducing the model easier. However, several studies do not provide information on the inputs, outputs, variables or detailed structures of their machine learning algorithms. In addition, over half of the studies^22 23 27–30^ were performed manually at the ROI extraction stage, and the machine learning algorithms in these studies may have required skilled expert radiologists, which is the ultimate limitation on utility. Other limitations include that only one study (Bordner *et al*^21^) reported missing data, and no study presented information on privacy measures, except for one study (Bressem *et al*^26^).

In our study, the number of publications was small because machine learning is a recently emerging technology. Even with this small number of studies, we performed a meta-analysis of the sensitivity and specificity values. However, significant heterogeneity was observed among the studies; therefore, we could not present a summary estimate based on the meta-analysis. The meta-analysis could have been conducted for the AUC or likelihood ratio (LR). However, the interpretation of a meta-analysis of the AUC or LR, which summarises values that do not consider the recall-precision trade-off (or threshold effect of the sensitivity and specificity) is controversial, and integrated estimates have been criticised for having a lower clinical utility than sensitivity and specificity.^20^ Furthermore, our study used PROBAST, which can be used for prediction models, including machine learning algorithms. However, PROBAST is a quality assessment tool primarily used for regression models; therefore, signalling questions that only apply to machine learning were considered, limiting the quality assessment.^13 14^ However, this problem will be solved using a quality assessment tool in the future because quality assessment tools customised for machine learning and AI models remain in development.^32^

Despite these limitations, we make the following recommendations to researchers and peer reviewers. We recommend adherence to AI-specific reporting and review guidelines. For example, the CLAIM^18^ ensures transparent reporting and a rigorous peer review for reproducibility and transparency purposes. In addition, owing to the small sample sizes involved, machine learning studies in specialty imaging, such as MRI, should be conducted in collaboration across countries and institutions. The active utilisation of common data elements,^18 33^ which are preformed reading and image-reporting systems, adds reproducibility and transparency to AI research. On the premise that researchers should report their findings transparently, we suggest an example of an ideal scenario to guide future research. As machine learning has a high number of events per variable, the optimal sample size usually exceeds 200 to prevent overfitting.^34^ Outcomes should be presented in detail in the form of metrics, not just representative values, as this is clinically useful.^35^ Owing to the complexity of MRI interpretations, the use of deep-learning techniques and their architectures is preferred.^36^ Finally, an external validation process with a completely independent population is usually recommended.^10^ However, given the high diversity and sophistication of machine learning techniques and the rare nature of rheumatic diseases, the ideal approach requires individualised tailoring for each study and situation.

## Conclusion

The performances of machine learning algorithms in discriminating MRI sacroiliitis features are highly heterogeneous across studies and experience problems with small sample sizes, overfitting risks and under-reporting issues, making conclusions and comparisons difficult. Future studies should encourage transparent reporting and study designs.

## Data Availability

Data are available on reasonable request. The raw data used for collection, extraction, and analysis is provided in a secure data-sharing environment. Requests will be reviewed on an individual basis. For more information about requests, please contact the corresponding author, HC (hoonsuk.cha@samsung.com).
