# Supplementary material for: Performances of machine learning algorithms in discriminating sacroiliitis features on MRI: a systematic review
Source: RMD Open. 2023 Nov 23;9(4):e003783. doi: 10.1136/rmdopen-2023-003783 (PMC10668284; doi:10.1136/rmdopen-2023-003783)
Supplement: Supplementary data [file rmdopen-2023-003783supp001.pdf]

Supplementary Fig. S1. Flow chart of screening process.

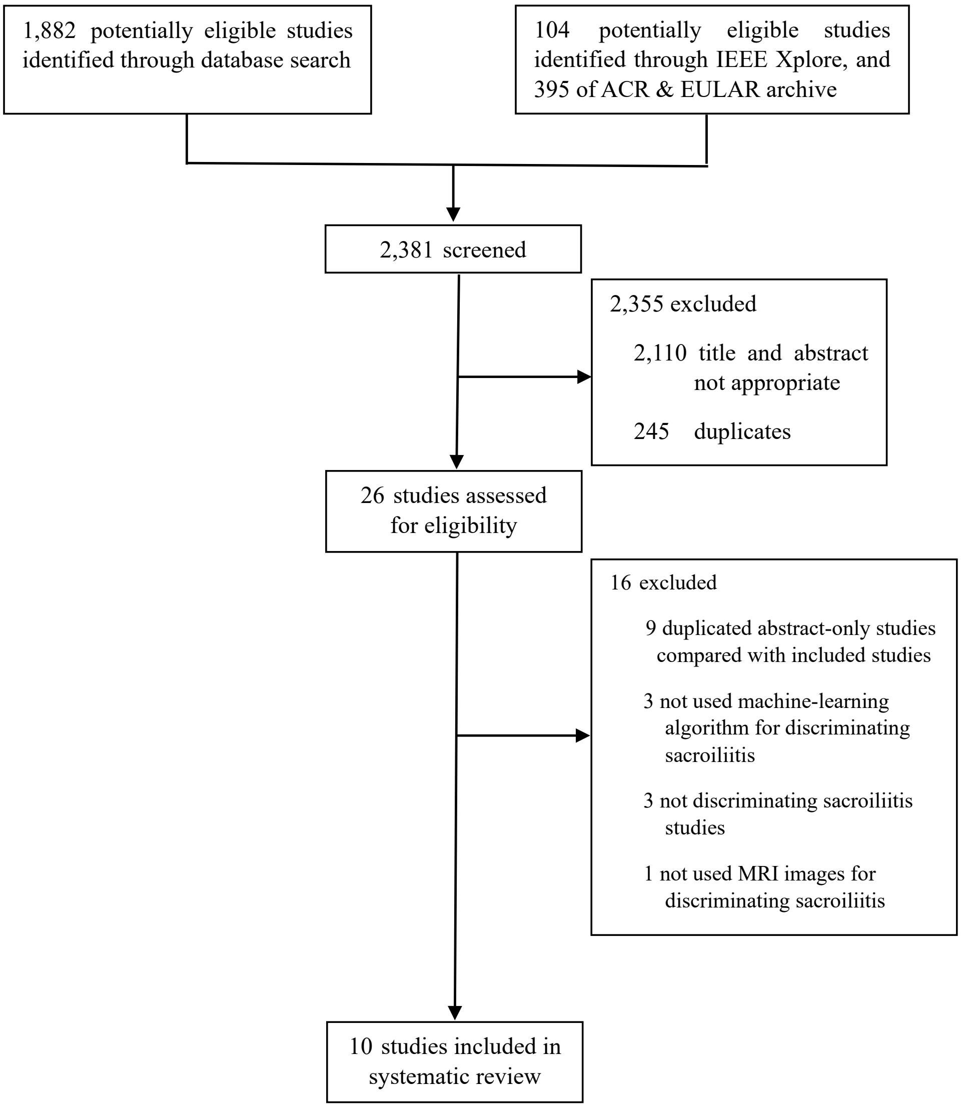

Supplementary Table S1. Search queries and search results.

	Embase & MEDLINE Complete (2023.06.04 GMT 14:35)	
#1	'spondyloarthropathy'/exp OR 'axial spondyloarthritis'/exp OR 'ankylosing spondylitis'/exp OR 'non-radiographic axial spondyloarthritis'/exp OR 'sacroiliac joint'/exp OR 'sacroiliitis'/exp OR spondyl* OR spondylarthr* OR spondyloarthropat* OR ankylos* OR sacroili* OR SI-joint* OR axspa OR nr-axspa	140,695
#2	'machine learning'/exp OR ‘machine-learning*’ OR ‘deep-learning*’ OR ‘neural-network*’ OR ‘automated-pattern-recognition*’ OR ‘back-propagate*’ OR ‘Bayesian*’ OR ‘Bayes*’ OR ‘classification-and-regression-tree*’ OR ‘classification-regression-tree*’ OR ‘classifier’ OR ‘classifiers’ OR ‘computer-heuristic*’ OR ‘confusion-matrix*’ OR ‘cross-validat*’ OR ‘data-mining*’ OR ‘feature-detect*’ OR ‘feature-extract*’ OR ‘feature-learn*’ OR ‘feature-rank*’ OR ‘feature-select*’ OR ‘fuzzy*’ OR ‘Markov*’ OR ‘iterative-closest*’ OR ‘iterative-dichotomiser*’ OR ‘k-means*’ OR ‘k-medians*’ OR ‘kernel-method*’ OR ‘least-absolute-shrinkage*’ OR ‘memristor*’ OR ‘multicriteria-decision*’ OR ‘multifactor-dimensionality-reduct*’ OR ‘network-learn*’ OR ‘online-analytical-process*’ OR ‘outlier-detect*’ OR ‘radial-basis-funct*’ OR ‘gradient-boosted-regression-tree*’ OR ‘random-forest*’ OR ‘recursive-feature-eliminat*’ OR ‘recursive-partition*’ OR ‘relevance-vector-machine*’ OR ‘rough-set*’ OR ‘semi-supervised*’ OR ‘supervised-machine*’ OR ‘support-vector*’ OR ‘unsupervised-machine*’ OR ‘regression-algorithm*’ OR ‘ordinary-least-squares-regression*’ OR ‘stepwise-regression*’ OR ‘multivariate-adaptive-regression-splines*’ OR ‘locally-estimated-scatterplot-smoothing*’ OR ‘instance-based*’ OR ‘nearest-neighbor*’ OR ‘learning-vector-quantization*’ OR ‘self-organizing-map*’ OR ‘locally-weighted-learning*’ OR ‘regularization-algorithm*’ OR ‘ridge-regression*’ OR ‘least-absolute-shrinkage-and-selection-operator*’ OR ‘least-absolute-shrinkage-selection-operator*’ OR ‘elastic-net*’ OR ‘least-angle-regression*’ OR ‘decision-tree*’ OR ‘chi-squared-automatic-interaction-detection*’ OR ‘decision-stump*’ OR ‘clustering-algorithm*’ OR ‘expectation-maximization*’ OR ‘hierarchical-clustering*’ OR ‘association-rule-learning-algorithm*’ OR ‘Apriori*’ OR ‘Eclat*’ OR ‘perceptron*’ OR ‘Hopfield-network*’ OR ‘radial-basis-function-network*’ OR ‘deep-Boltzman-machine*’ OR ‘belief-network*’ OR ‘auto-encoder*’ OR ‘dimensionality-reduction-algorithm*’ OR ‘ensemble-algorithm*’ OR ’bootstrapped-aggregation*’ OR ‘Adaboost*’ OR ‘stacked-generalization*’ OR ‘gradient-boosting-machine*’ OR ‘case-based-reasoning*’ OR ‘simulated-annealing*’ OR ‘inductive-logical-program*’ OR ‘genetic-algorithm*’	727,709
#3	'area under the curve'/mj OR ‘sensitivity and specificity’/mj OR 'receiver operating characteristic'/mj OR 'diagnostic accuracy'/mj OR ‘Youden-index*’ OR ‘likelihood-ratio*’ OR ‘diagnostic-odds-ratio*’ OR ‘area-under-the-curve*’ OR ‘area-under-curve*’ OR ‘receiver-operating-characteristic*’ OR ‘sensitivity*’ OR ‘specificity*’ OR ‘positive-predictive-value*’ OR ‘negative-predictive-value*’ OR ‘true-positive*’ OR ‘false-positive*’ OR ‘true-negative*’ OR ‘false-negative*’ OR ‘four-by-four’ OR ‘4-by-4’ OR accura* OR ‘ROC’ OR ‘AUC’	3,791,611
#4	#1 AND #2 AND #3	570
#5	#4 AND [2008-2023]/py	552
Database	CINAHL Complete (2023.06.04 GMT 14:42)	
#1	(MH "Spondylarthropathies+") OR (MM "Axial Spondyloarthritis+") OR (MH "Non-Radiographic Axial Spondyloarthritis") OR (MH "Spondylitis, Ankylosing") OR (MH "Sacroiliac Joint") OR (MH "Sacroiliitis") OR spondyl* OR spondylarthr* OR spondyloarthropat* OR ankylos* OR sacroili* OR SI-joint* OR axspa OR nr-axspa	27,680
#2	(MH "Machine Learning+") OR machine-learning* OR deep-learning* OR neural-network* OR automated-pattern-recognition* OR back-propagate* OR Bayesian* OR Bayes* OR classification-and-regression-tree* OR classification-regression-tree* OR classifier OR classifiers OR computer-heuristic* OR confusion-matrix*	116,303

	OR cross-validat* OR data-mining* OR feature-detect* OR feature-extract* OR feature-learn* OR feature-rank* OR feature-select* OR fuzzy* OR Markov* OR iterative-closest* OR iterative-dichotomiser* OR k-means* OR k-medians* OR kernel-method* OR least-absolute-shrinkage* OR memristor* OR multicriteria-decision* OR multifactor-dimensionality-reduct* OR network-learn* OR online-analytical-process* OR outlier-detect* OR radial-basis-funct* OR gradient-boosted-regression-tree* OR random-forest* OR recursive-feature-eliminat* OR recursive-partition* OR relevance-vector-machine* OR rough-set* OR semi-supervised* OR supervised-machine* OR support-vector* OR unsupervised-machine* OR regression-algorithm* OR ordinary-least-squares-regression* OR stepwise-regression* OR multivariate-adaptive-regression-splines* OR locally-estimated-scatterplot-smoothing* OR instance-based* OR nearest-neighbor* OR learning-vector-quantization* OR self-organizing-map* OR locally-weighted-learning* OR regularization-algorithm* OR ridge-regression* OR least-absolute-shrinkage-and-selection-operator* OR least-absolute-shrinkage-selection-operator* OR elastic-net* OR least-angle-regression* OR decision-tree* OR chi-squared-automatic-interaction-detection* OR decision-stump* OR clustering-algorithm* OR expectation-maximization* OR hierarchical-clustering* OR association-rule-learning-algorithm* OR Apriori* OR Eclat* OR perceptron* OR Hopfield-network* OR radial-basis-function-network* OR deep-Boltzman-machine* OR belief-network* OR auto-encoder* OR dimensionality-reduction-algorithm* OR ensemble-algorithm* OR bootstrapped-aggregation* OR Adaboost* OR Stacked-generalization* OR gradient-boosting-machine* OR case-based-reasoning* OR simulated-annealing* OR inductive-logical-program* OR genetic-algorithm*	
#3	(MH "ROC Curve") OR (MH "Sensitivity and Specificity") OR Youden-index* OR likelihood-ratio* OR diagnostic-odds-ratio* OR area-under-the-curve* OR area-under-curve* OR receiver-operating-characteristic* OR sensitivity* OR specificity* OR positive-predictive-value* OR negative-predictive-value* OR true-positive* OR false-positive* OR true-negative* OR false-negative* OR four-by-four OR 4-by-4 OR accura* OR AUC OR ROC	820,180
#4	#1 AND #2 AND #3	678
#5	#4 AND [2008-2023]/py	634
Database	Web of Science (2023.06.04 GMT 15:03)	
#1	ALL=(spondyl* OR spondylarthr* OR spondyloarthropat* OR ankylos* OR sacroili* OR SI-joint* OR axspa OR nr-axspa)	55,854
#2	ALL=(learn* OR train* OR validat* OR learning OR training OR validating OR machine OR deep OR neural-network* OR automated-pattern-recognition* OR back-propagate* OR Bayesian* OR Bayes* OR classification-and-regression-tree* OR classification-regression-tree* OR classifier OR classifiers OR computer-heuristic* OR confusion-matrix* OR feature-detect* OR feature-extract* OR feature-learn* OR feature-rank* OR feature-select* OR fuzzy* OR Markov* OR iterative-closest* OR iterative-dichotomiser* OR k-means* OR k-medians* OR kernel-method* OR least-absolute-shrinkage* OR memristor* OR multicriteria-decision* OR multifactor-dimensionality-reduct* OR network-learn* OR online-analytical-process* OR outlier-detect* OR radial-basis-funct* OR gradient-boosted-regression-tree* OR random-forest* OR recursive-feature-eliminat* OR recursive-partition* OR relevance-vector-machine* OR rough-set* OR semi-supervised* OR supervised-machine* OR support-vector* OR unsupervised-machine* OR regression-algorithm*)	4,546,580
#3	ALL=(Youden-index* OR likelihood-ratio* OR diagnostic-odds-ratio* OR area-under-the-curve* OR area-under-curve* OR receiver-operating-characteristic* OR sensitivity* OR specificity* OR positive-predictive-value* OR negative-predictive-value* OR true-positive* OR false-positive* OR true-negative* OR false-negative* OR four-by-four OR 4-by-4 OR accura* OR AUC OR ROC)	3,535,292
#4	#1 AND #2 AND #3	788
#5	#4 AND [2008-2023]/py	696
Database	IEEE Xplore digital library	
#1	(spondyloarthropathy OR spondyloarthritis OR ankylosing OR spondylitis OR	

	sacroiliitis OR scroiliitis OR SI-joint OR axspa) AND (Youden index OR diagnostic odds ratio OR area under the curve OR area under curve OR AUC OR receiver-operating-characteristic OR ROC OR sensitivity OR specificity OR positive predictive value OR negative predictive value OR true positive OR false positive true negative OR false negative OR accuracy OR accuracies OR AUC)	
#2	#1 AND [2008-2023]/py	104
Database	ACR and EULAR archive	
	machine learning OR deep learning	395

ACR: American College of Rheumatology; CINAHL: Cumulative Index to Nursing and Allied Health Literature; EULAR: European Alliance of Associations for Rheumatology; GMT: Greenwich Mean Time; IEEE: Institute of Electrical and Electronics Engineers;

Supplementary Table S2. Clinical characteristics of the included studies (five studies).

	Symptom duration, mean years	Mean ESR, mm/h	Mean CRP, mg/dl	HLA-B27, positivity (%)
Bordner *et al.* [21]	1.60 for DESIR cohort and 9.00 for ASAS cohort	Not reported	0.66 for DESIR cohort and 0.36 for ASAS external cohort	158/256 (62%) for DESIR cohort and 26/47 (55%) ASAS cohort
Ye *et al.* [22]	3.10 for training cohort and 3.11 for validation cohort	30.60 for training cohort and 31.90 for validation cohort	2.51 for training cohort and 2.25 for validation cohort	333/447 (75%) for training cohort and 137/191 (72%) for validation cohort
Tenório *et al.* [23]	Not reported	13.27 for baseline cohort	1.53 for baseline cohort	Not reported for baseline cohort
Lin *et al.* [25]	11.60 for SpA group and 8.80 for NSBP group	32.00 for training/validation group and 32.90 for internal test cohort	1.00 for training/validation cohort and 1.10 for internal test cohort	234/329 (71%) for training/validation cohort and 21/28 (75%) for internal test cohort
Bressem *et al.* [26]	7.00 to 13.00 for training/validation cohort and 8.00 for external test cohort	Not reported	0.40 to 1.30 for training/validation cohort and 0.33 for external test cohort	296/477 (63%) for training/validation cohort and 47/116 (41%) for external test cohort

ASAS: Assessment of Spondyloarthritis International Society; CRP: C-reactive protein; DESIR: DEvenir des Spondyloarthropathies Indifferenciees Recentes; ESR: erythrocyte sedimentation rate; NSBP: non-specific back pain; HLA-B27: Human leukocyte antigen-B27; SpA: spondyloarthritis.

Supplementary Table S3. Performance of validation process of machine learning in detail (eight studies).

	Ground truth	Validation technique	Performance of classification
Ye *et al.* [22]	AxSpA +/-	Split-validation	AUC of validation process was 0.90 (95% CI: 0.85, 0.94). The model yielded 0.71 sensitivity, 0.81 specificity, 0.88 positive predictive value, 0.58 negative predictive value, and 0.74 (0.68-0.80) accuracy.
Tenório *et al.* [23]	ASAS MRI sacroiliitis +/-	Cross-validation	AUC of validation process ranged 0.86 to 0.88. The model yielded 0.75 (95% CI: 0.51-0.90) sensitivity and 0.85 (0.65-0.95) specificity with the STIR sequence and 1.00 (0.80-1.00) sensitivity and 0.89 (0.70-0.97) specificity with the SPAIR sequence.
Roel *et al.* [24]	BME +/-	Cross-validation	AUC of validation process was 0.95. The model yielded 0.82 balanced accuracy and 0.62 F1 score.
Bressem *et al.* [26]	ASAS MRI sacroiliitis +/-	Split-validation	AUC of validation process of active inflammatory changes, ASAS-compatible changes, and structural changes were 0.90, 0.92, and 0.86. Sensitivity of active inflammatory changes, ASAS-compatible changes, and structural changes were 0.96, 0.75, and 0.95. Specificity of active inflammatory changes, ASAS-compatible changes, and structural changes were 0.76, 0.86, and 0.75. Accuracy of active inflammatory changes, ASAS-compatible changes, and structural changes were 0.84, 0.82, and 0.85.
Nicolaes *et al.* [27]	BME +/-	Cross-validation	AUC of validation process was 0.77. The model yielded 0.70 sensitivity, 0.74 specificity, and 0.79 precision.
Lee *et al.* [28]	ASAS MRI sacroiliitis +/-	Cross-validation	AUC of validation process ranged 0.97 to 0.99. The model yielded 0.93 recall, 0.94 specificity, 0.95 precision, 0.93 negative predictive value, and 0.94 accuracy (per images). The model yielded 1.00 recall, 0.86 specificity, 0.95 precision, 1.00 negative predictive value, and 0.96 accuracy (per MRI).
Kepp *et al.* [29]	ASAS MRI sacroiliitis +/-	Cross-validation	Overall machine learning AUC for the determination of SpA was 0.92.
Faleiros *et al.* [30]	ASAS MRI sacroiliitis +/-	Cross-validation	AUC of validation process ranged 0.80 to 0.97. MLP classifier obtained the best performance with sensitivity 1.00, specificity 0.96, and accuracy 0.85.

ASAS: Assessment of Spondyloarthritis International Society; AUC: area under the curve; AxSpA: axial spondyloarthritis; BME: bone marrow edema; CI: confidence interval; MCC: Matthews correlation coefficient; MLP: multilayer perceptron; MRI: Magnetic Resonance Imaging.

Supplementary Table S4. Performance of internal test process of machine learning in detail (three studies).

	Ground truth	Performance of classification
Bordner *et al.* [21]	ASAS MRI sacroiliitis +/-	AUC of internal test ranged 0.80 to 0.98. The model yielded 0.80 sensitivity (95% CI: 0.64-0.90) and 0.88 specificity (0.81-0.92). At baseline, the MCC between the machine learning model and the majority decision for the determination of MRI ASAS status was 0.90.
Lin *et al.* [25]	ASAS MRI sacroiliitis +/-	AUC of internal test process of original dataset and the fake-color image dataset were 0.92 and 0.96. The model of the original dataset (per images) yielded 0.86 sensitivity and 0.92 specificity, fake-color image (per images) dataset yielded 0.90 sensitivity and 0.93 specificity. The model of the original dataset (per MRI) yielded 0.94 sensitivity and 0.79 specificity, fake-color image (per MRI) dataset yielded 0.94 sensitivity and 0.95 specificity.
Faleiros *et al.* [30]	ASAS MRI sacroiliitis +/-	AUC of test process was 0.92. MLP classifier obtained sensitivity 1.00, specificity 0.67, and accuracy 0.80.

ASAS: Assessment of Spondyloarthritis International Society; AUC: area under the curve; CI: confidence interval; MLP: multilayer perceptron; MRI: Magnetic Resonance Imaging.

Supplementary Table S5. Performance of external test process of machine learning in detail (three studies).

	Ground truth	Performance of classification
Bordner *et al.* [21]	ASAS MRI sacroiliitis +/-	AUC of external test process was 0.76 (0.57-0.95). Model yielded 0.56 sensitivity (95% CI: 0.42-0.70), 1.00 specificity (1.00-1.00), 0.81 accuracy (0.88-1.00). MCC between the machine learning model and the majority decision for the determination of MRI ASAS status was 0.62.
Roel *et al.* [24]	BME +/-	AUC of external process was 0.88. The model yielded 0.72 balanced accuracy and 0.51 F1 score.
Bressem *et al.* [26]	ASAS MRI sacroiliitis +/-	AUC of validation process of active inflammatory changes, ASAS-compatible changes, and structural changes were 0.94, 0.86, and 0.89. Sensitivity of active inflammatory changes, ASAS-compatible changes, and structural changes were 0.88, 0.86, and 0.85. Specificity of active inflammatory changes, ASAS-compatible changes, and structural changes were 0.71, 0.76, and 0.78. Accuracy of active inflammatory changes, ASAS-compatible changes, and structural changes were 0.75, 0.78, and 0.79.

ASAS: Assessment of Spondyloarthritis International Society; AUC: area under the curve; AxSpA: axial spondyloarthritis; BME: bone marrow edema; CI: confidence interval; MRI: Magnetic Resonance Imaging.

Supplementary Table S6. Quality assessment in detail (ten studies).

	Risk of bias<br>Participants	Predictors	Outcome	Analysis	Applicability<br>Participants	Predictors	Outcome
Bordner *et al.* [21]	Low	Low	Low	High	Low	Low	Low
Ye *et al.* [22]	Low	Low	Low	High	Low	Low	Low
Tenório *et al.* [23]	Unclear	Low	Low	High	Unclear	Low	Low
Roel *et al.* [24]	Unclear	Unclear	Unclear	Unclear	Unclear	Unclear	Low
Lin *et al.* [25]	Low	Low	Low	High	Low	Low	Low
Bressem *et al.* [26]	Low	Low	Low	Low	Low	Low	Low
Nicolaes *et al.* [27]	Unclear	Unclear	Unclear	Unclear	Unclear	Unclear	Low
Lee *et al.* [28]	Unclear	Low	Low	High	Unclear	Low	Low
Kepp *et al.* [29]	Low	Low	Low	High	Low	Low	Low
Faleiros *et al.* [30]	Unclear	Low	Low	High	Unclear	Low	Low
